# PEDF inhibits pancreatic tumorigenesis by attenuating the fibro-inflammatory reaction

**DOI:** 10.18632/oncotarget.8587

**Published:** 2016-04-05

**Authors:** Daniel R. Principe, Brian DeCant, Andrew M. Diaz, Riley J. Mangan, Rosa Hwang, Andrew Lowy, Brandon B. Shetuni, Bharath K. Sreekumar, Chuhan Chung, David J. Bentrem, Hidayatullah G. Munshi, Barbara Jung, Paul J. Grippo, Faraz Bishehsari

**Affiliations:** ^1^ University of Illinois College of Medicine, Champaign, IL, USA; ^2^ Department of Medicine, University of Illinois at Chicago, Chicago, IL, USA; ^3^ Department of Surgical Oncology, Division of Surgery, The University of Texas MD Anderson Cancer Center, Houston, TX, USA; ^4^ Department of Surgery, University of California San Diego, San Diego, CA, USA; ^5^ Northwestern Medicine, Central DuPage Hospital, Winfield, IL, USA; ^6^ Department of Medicine, Yale University School of Medicine, New Haven, CT, USA; ^7^ Robert H. Lurie Comprehensive Cancer Center, Feinberg School of Medicine, Northwestern University, Chicago, IL, USA; ^8^ Department of Medicine, Rush University Medical Center, Chicago, IL, USA

**Keywords:** pancreatic cancer, PEDF, inflammation, fibrosis, KRAS

## Abstract

Pancreatic cancer is characterized by a pronounced fibro-inflammatory reaction that has been shown to contribute to cancer progression. Previous reports have demonstrated that pigment epithelium-derived factor (PEDF) has potent tumor suppressive effects in pancreatic cancer, though little is known about the mechanisms by which PEDF limits pancreatic tumorigenesis. We therefore employed human specimens, as well as mouse and *in vitro* models, to explore the effects of PEDF upon the pancreatic microenvironment. We found that PEDF expression is decreased in human pancreatic cancer samples compared to non-malignant tissue. Furthermore, PEDF-deficient patients displayed increased intratumoral inflammation/fibrosis. In mice, genetic ablation of PEDF increased cerulein-induced inflammation and fibrosis, and similarly enhanced these events in the background of oncogenic KRAS. *In vitro*, recombinant PEDF neutralized macrophage migration as well as inhibited macrophage-induced proliferation of tumor cells. Additionally, recombinant PEDF suppressed the synthesis of pro-inflammatory/pro-fibrotic cytokines both *in vivo* and *in vitro*, and reduced collagen I deposition and TGFβ synthesis by pancreatic stellate cells, consistent with reduced fibrosis. Combined, our results demonstrate that PEDF limits pancreatic cancer progression by attenuating the fibro-inflammatory reaction, and makes restoration of PEDF signaling a potential therapeutic approach to study in pancreatic cancer.

## INTRODUCTION

The causal link between inflammation and cancer has been proposed for over a century [1]. Recently, mechanisms by which chronic inflammation promotes different types of human cancers are being recognized [2, 3]. This also applies to pancreatic cancer, where prominent fibrosis accompanies both developing and invasive cancer. Pancreatic ductal adenocarcinoma (PDAC) is the most fatal of all cancers and the fourth leading cause of cancer-related deaths in the United States. Its dismal ∼7-8% 5-year survival has remained relatively unchanged for decades. In fact, the death rate from pancreatic cancer per 100,000 has increased an average of 0.4% each year between 2002 and 2011[4]. Therefore, to design more effective treatments, a better understanding of the mechanisms regulating pancreatic cancer progression, including the role of the tumor microenvironment (TME), is needed.

A large body of evidence suggests that tumor-associated inflammation and fibrosis are key events in pancreatic cancer etiology [5–7]. This is substantiated clinically, as it is well established that patients with a history of chronic pancreatitis have a higher risk of subsequent pancreatic cancer development [8–10]. Furthermore, human pancreatic cancers have a robust immune cell infiltrate and presence of inflammatory cells linked to enhanced local and distant metastasis, worse tumor stage, and reduced overall survival [5, 6, 11]. This immune response is characterized by decreased anti-tumor cytotoxicity and increased pro-tumorigenic immune components including tumor-associated macrophages which have been implicated in enhancing both tumor proliferation and chemoresistance [12–17]. Furthermore, pancreatic cancer-associated inflammation is associated with a dense desmoplastic reaction indicative of matrix deposition [18]. This is mediated by pancreatic stellate cells (PSCs) and dependent on growth factors such as TGFβ [19], which are likely to play a pivotal role in mediating this type of pancreatic fibrosis [20, 21]. In human PDAC tumor tissue, there are focal areas of fibrosis with elevated TGFβ signaling, which may further promote tumor progression [22–25]. Yet, the molecular mechanism(s) and players involved in this underlying pro-tumorigenic, fibro-inflammatory response are not well understood.

Recent evidence has substantiated the notion that Pigment Epithelium-Derived Factor (PEDF) contributes to regulating the inflammatory response, with more studies suggesting anti-inflammatory [26, 27] and tumor suppressor roles for PEDF, with strong therapeutic potential in a variety of tumor types [28–30]. PEDF is a non-inhibitory serpin family member with potent neurotrophic and anti-angiogenic effects. While these effects have been well-characterized, other molecular events underlying its anti-inflammatory function remain unclear. PEDF signals through multiple high affinity ligands and receptors, including interactions with adipose triglyceride lipase (ATGL) [31], perhaps the most well understood identified PEDF receptor. However, it has also been observed that mice with genetic ablation of either ATGL or PEDF are phenotypically distinct with respect to abnormal prostate and eye development [32–34], further substantiating the possibility that PEDF may signal through several non-ATGL pathways. More recent work identifies the effects of PEDF on inflammatory as well as carcinogenic signals may well be mediated through ATGL while other receptors (such as laminin receptors, and membrane protein F1 ATP synthase) are localized in the endothelial cells and more closely linked to the anti-angiogenic functions of PEDF [31, 35–37].

While the anti-angiogenic functions of PEDF continue to be well-studied, the role of PEDF in other cell types, particularly in inflammatory, mesenchymal, and cancer cells, is only recently becoming more clear. In the pancreas, PEDF is expressed by both ductal and acinar cells [38] and was first shown to regulate the mass and vascularity of the pancreas [33]. In mice, genetic ablation of PEDF leads to enhanced cerulein-induced pancreatitis and poorer recovery, with enhanced early fibrotic effects mediated by TGFβ1 [39]. Stable overexpression of PEDF in epithelial cells inhibited the development of subcutaneous tumors in xenograft models [40]. Furthermore, clinical observations suggest that PEDF expression correlates with both increased survival time and reduced metastasis in PDAC patients [41].

We previously have demonstrated that genetic ablation of PEDF in mice leads to enhanced cerulein-induced pancreatitis and associated fibrosis [39]. Indeed, PEDF has been shown to be down-regulated in several inflammation-associated cancers including lung, prostate, breast, and pancreatic [28]. PEDF expression correlates with both increased survival time and reduced metastasis in PDAC patients [41]. In an autochthonous model of pancreatic cancer, we have previously shown that loss of PEDF results in accelerated mutant KRAS-induced pancreatic tumorigenesis, concomitant with metastatic disease [42]. These studies strongly suggest that PEDF has tumor suppressor activity in pancreatic cancer, though the mechanisms by which PEDF limits pancreatic tumorigenesis is less known. While anti-angiogenic functions of PEDF have been best characterized, down regulation of PEDF in a poorly vascularized tumor such as pancreatic cancer [43–45] suggests other potential anti-tumoral mechanisms for PEDF in this context. Here, we propose that loss of PEDF affects the TME and ultimately tumorigenesis by enhancing inflammation and fibrosis in the pancreas. Using human tissue and a range of *in vivo* and *in vitro* models, we provide evidence in favor of our hypothesis.

## RESULTS

### PEDF expression is decreased in human pancreatic cancer and inversely correlates with inflammation

**Figure 1:**
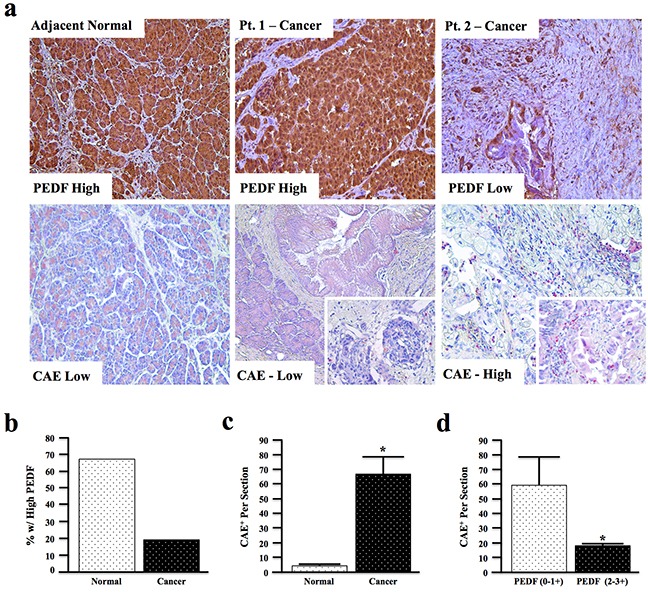
PEDF expression is decreased in human pancreatic cancer and inversely correlates with tissue inflammatory profile **a.** Human pancreatic cancer and adjacent normal tissue sections (n=66) were stained for PEDF and scored from 0-3^+^, or for Chloracetate Esterase (CAE) activity and the number of CAE^+^ positive cells per 10x field quantified (pictures at 200X and two inserts at 400X). **b.** The percentage of sections with high PEDF expression in adjacent normal and cancer specimens was examined. **c.** CAE^+^ cells in adjacent normal and cancer sections were quantified per 10x field. **d.** The correlation between PEDF score and CAE^+^ cells per section in cancer tissue was analyzed by two-way ANOVA. (*, p<0.05).

To assess the relationship between PEDF and pancreatic cancer-associated inflammation, human pancreatic cancer and adjacent non-malignant sections (N=66) were stained for PEDF by immunohistochemistry. Sections were then independently scored from 0-3+ based on intensity by two blinded investigators. Serial sections were also stained chemically for Chloracetate Esterase (CAE), which identifies mast cells, neutrophils, and other inflammatory granulocytes [46]. The number of CAE^+^ cells was then quantified per high power field, also by two blinded investigators (Figure 1a).

In this patient cohort, approximately 70% of the non-malignant pancreas samples had high (3+) PEDF staining in both acinar and ductal cells (Figure 1a), while only ∼20% of cancer sections demonstrated high PEDF staining (Figure 1b). Conversely, fewer than 5% of non-malignant samples had undetectable PEDF staining while nearly 30% of cancer sections had complete loss of PEDF (Supplementary Figure S1a).

In addition to having significantly reduced PEDF expression (Supplementary Figure S1b, p<0.01), malignant sections also displayed significantly increased CAE^+^ cell infiltration (Figure 1c, p<0.0001). Interestingly, within the cancer tissue, sections with low PEDF staining (0-1+) demonstrated significantly more CAE^+^ cells than tumors with high PEDF scores (2-3+) (Figure 1d, p<0.05), indicating that PEDF inversely correlates with tumor-associated inflammation in the examined cohort.

### Loss of PEDF enhances cerulein-induced inflammation and fibrosis

Our group has previously shown that genetic ablation of PEDF was permissive for increased TGFβ1 and collagen expression in mouse pancreas. We have also shown that PEDF expression increases in response to cerulein-induced pancreatitis, and PEDF^−/−^ animals display prominent increases in stellate cell-mediated fibrosis [39]. The effects of PEDF deficiency on pancreatitis-associated inflammation are not known. To determine whether the loss of PEDF is permissive for increased pancreatic inflammation *in vivo*, we evaluated the effects of acute cerulein-induced pancreatitis in control and PEDF-null (PEDF^−/−^) mice.

**Figure 2:**
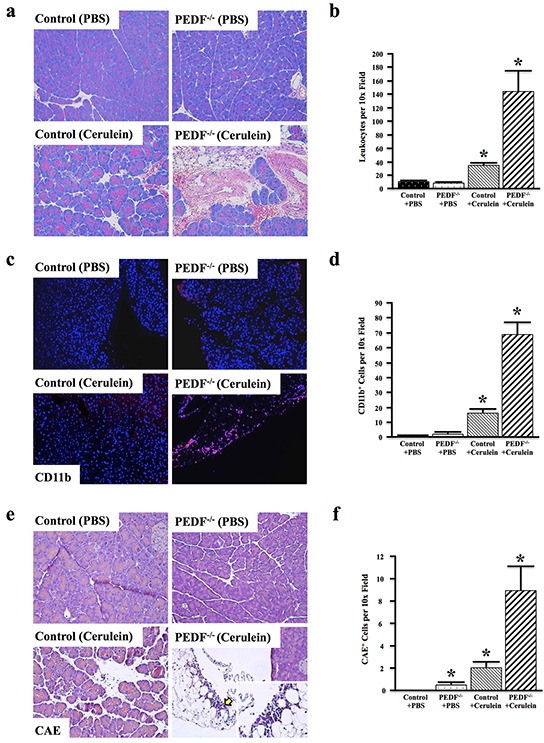
Loss of PEDF enhances cerulein-induced inflammation and fibrosis Control and PEDF^−/−^ mice (n=3/group) were generated as detailed in Materials and Methods, and treated with cerulein to induce acute pancreatitis. **a–b.** The pancreatic tissue sections were stained with hematoxylin and eosin (H&E), and the number of leukocytes per 10x field was quantified. **c–d.** Sections were stained for the pan-myeloid cell marker CD11b, and the number of CD11b^+^ cells per 10x field was quantified. **e–f.** CAE staining was done (yellow arrow denotes area of high CAE+ cell infiltrate)., and the number of CAE^+^ cells per 10x field was quantified. (*, p<0.05) (for all comparisons with Control + PBS). PEDF + Cerulein were all significantly higher than PEDF + PBS. For a, c, & e, all pictures at 100X and single insert at 400X).

Compared to *wild type* littermate controls, PEDF^−/−^ mice developed more severe pancreatitis that was associated with increased overall leukocyte infiltration and tissue damage (Figure 2a, 2b). Similarly, PEDF^−/−^ mice treated with cerulein also displayed increased CD11b^+^ myeloid cell infiltration (Figure 2c, 2d), particularly in areas with more severe tissue damage (Figure 2c, lower right). In accordance with these observations, PEDF^−/−^ mice also presented with increased CAE^+^ cell infiltration in response to cerulein (Figure 2e, 2f), suggesting an increased myeloid-derived immune cell infiltration in the pancreas, particularly in areas of severe damage (Figure 2e, lower right). Together, these data suggest that the loss of PEDF promotes greater inflammation in the pancreas upon induction.

### PEDF deficiency accelerates tumor-associated inflammation *in vivo*

**Figure 3:**
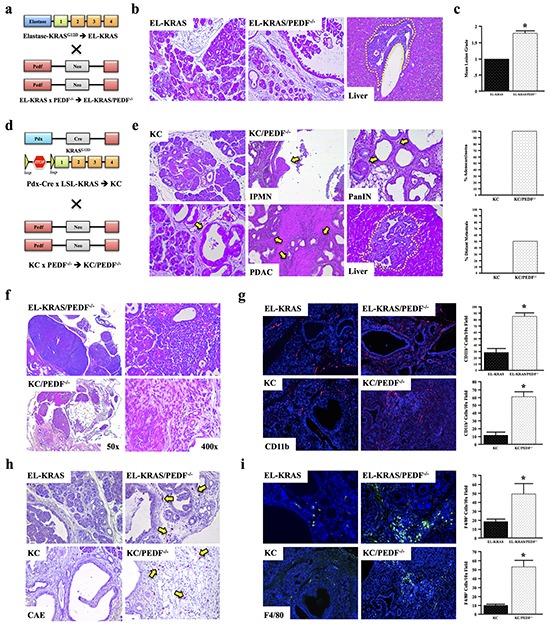
PEDF deficiency accelerates tumor-associated inflammation in vivo **a–c.** Mice expressing mutant KRAS^G12D^ were crossed to PEDF^−/−^ mice to generate EL-KRAS/PEDF^−/−^ animals (n=4/group). Pancreatic tumor sections from control KRAS^G12D^ mice and EL-KRAS/PEDF^−/−^ mice were stained with hematoxylin and eosin (H&E), and the sections graded from 0-3+. **d, e.** Pdx-Cre x LSL-KRAS (KC) mice were crossed to PEDF^−/−^ animals to generate KC/ PEDF^−/−^ mice. The pancreas and liver were similarly stained with H&E and subject to pathological analyses. **f.** Tissue sections from EL-KRAS, EL-KRAS/PEDF^−/−^ (n=4/group), KC, and KC/PEDF^−/−^ (n=3/group) were analyzed for lymphadenopathy and tumor-associated infiltration in the pancreas. **g–i.** Tissue sections were next stained for the myeloid markers CD11b, CAE, and for the macrophage marker F4/80. (*, p<0.05). (All pictures at 100X except for 3f at 50X and 400X).

To further evaluate the role of PEDF in tumor-associated inflammation, C57Bl/6 mice expressing mutant KRAS^G12D^ in the pancreas were crossed with PEDF^−/−^ mice (Figure 3a), consistent with our previous study. Tissues were collected after one year and reviewed by three blinded investigators. Compared to control EL-KRAS mice, EL-KRAS/PEDF^−/−^ mice developed larger and more poorly differentiated lesions (Figure 3b, 3c). Additionally, 2/10 of EL-KRAS/PEDF^−/−^ mice developed pancreatic ductal adenocarcinoma with distant liver metastases (Figure 3b), compared to 0/10 EL-KRAS control mice. In our prior work [42], we observed a 5% metastatic rate in EL-KRAS/PEDF^−/−^ mice in the FVB6 F2 background strain. Here, we extended our work entirely into the B6 strain and found an even more potent phenotype (metastatic rate of 20%), potentially due to yet unidentified modifiers in this background strain.

In the elastase-driven KRAS^G12D^ model, lesions resemble pre-cancerous human intraductal papillary mucinous neoplasms (IPMNs). Yet, the majority of PDAC patients develop cancer originating from pancreatic intraepithelial neoplasms (PanINs) [47]. Therefore, we next utilized the Pdx-Cre/LSL-KRAS^G12D^ (KC) model of PanIN disease (Figure 3d). KC mice were crossed to PEDF^−/−^ animals to generate KC/PEDF^−/−^ mice (Figure 3d, 3e). Similar to EL-KRAS/PEDF^−/−^ mice, 100% (4/4) of KC/PEDF^−/−^ mice presented with highly advanced neoplastic lesions, and locally invasive adenocarcinoma at seven months, with 50% having distant liver metastases (Figure 3e).

Both EL-KRAS/PEDF^−/−^ and KC/PEDF^−/−^ mice presented with peri-pancreatic lymphadenopathy and enhanced leukocyte infiltration, consistent with a more robust inflammatory response (Figure 3f). Furthermore, both EL-KRAS/PEDF^−/−^ and KC/PEDF^−/−^ mice had increased pancreas involvement of cells that stained positive for the pan-myeloid marker CD11b (Figure 3g), CAE (Figure 3h), and the mouse macrophage marker F4/80 (Figure 3i). These findings support the increase in macrophage-driven tumor-associated in the pancreas of PEDF^−/−^ mice.

### PEDF inhibits macrophage activation *in vitro*

**Figure 4:**
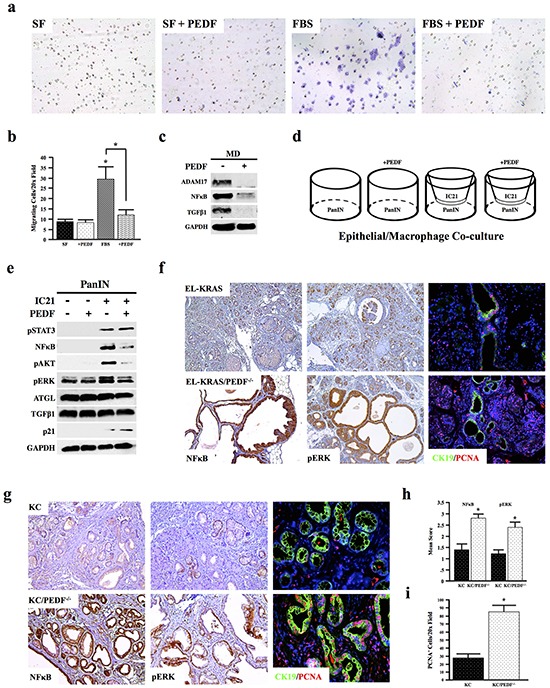
PEDF inhibits macrophage activation *in vitro* **a–b.** The effect of rPEDF on the ability of IC21 murine macrophage to migrate through 8-micron transwell filters over 24 hours was examined, and the number of migrating cells per 10x field was quantified. **c.** Human MD macrophages were incubated with 20ng/ml rPEDF for 24 hours, and the effects on ADAM17, NFkB, TGFβ1, and GAPDH were determined by Western blot analysis of the macrophage lysates. **d–e.** Co-cultures were established with murine neoplastic epithelial cells (PanIN) and IC21 macrophages. Co-cultures were incubated with 20ng/ml rPEDF over 24 hours and lysates from the PanIN cell line were subjected to Western blot analysis (*, p<0.05). **f–i.** Pancreatic tumor sections from EL-KRAS, EL-KRAS/PEDF^−/−^ (n=4/group), KC, and KC/PEDF^−/−^ (n=3/group) mice were stained for NFκB, pERK, or dual stained for the ductal marker CK19 and for proliferation marker PCNA. (4a pictures at 200X, 4f at 100X, & 4g at 200X).

Accumulating evidence shows a remarkable role for macrophages in pancreatic cancer progression [16]. Given the increased inflammation in both EL-KRAS/PEDF^−/−^ and KC/PEDF^−/−^ mice, we next examined whether PEDF directly affected macrophage migration *in vitro*. We found that incubation with recombinant PEDF neutralized migration of murine IC21 macrophages towards FBS (Figure 4a, 4b). Recombinant PEDF (rPEDF) also reduced expression of the pro-inflammatory protein marker, NFκB as well as ADAM17 in MD human macrophages, indicating reduced macrophage activation and potential interplay between PEDF and the NFκB cascade. In addition, PEDF decreased expression of TGFβ1 in MD macrophages (Figure 4c).

To extend these findings to pancreatic carcinogenesis, we established co-cultures of murine PanIN KC4848 neoplastic epithelial cells, isolated from the KC model [48], with IC21 murine macrophages (Figure 4d). When cultured alone, treatment with rPEDF had no observable effects on mitogenic signaling in epithelial cells. While co-culture of IC21 macrophages increased expression of mitogens pSTAT3, pAKT, NFκB, and pERK expression in PanIN epithelial cells, these responses, with the exception of pSTAT3, were inhibited by addition of exogenous PEDF. Consistent with increased cell-cycle arrest, PEDF treatment in the co-cultures also increased expression of the cyclin-dependent kinase inhibitor p21 in the epithelial cells (Figure 4e). In line with these *in vitro* findings, EL-KRAS/PEDF^−/−^ and KC/PEDF^−/−^ animals both displayed increased staining for NFκB and pERK and increased proliferation *in vivo* in both tissue undergoing acinar-ductal metaplasia (CK19^+^) and in normal acini (Figure 4f–4i).

### PEDF modifies inflammatory and fibrotic cytokines both *in vitro* and *in vivo*

**Figure 5:**
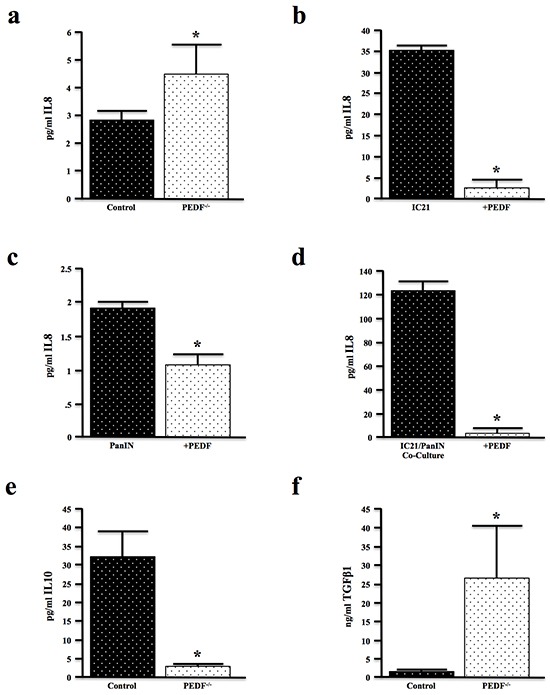
PEDF modifies inflammatory and fibrotic cytokines both *in vitro* and *in vivo* **a.** Serum samples from four-month-old control and PEDF^−/−^ mice (n=3/group) were evaluated for expression of the inflammatory cytokine IL8 by multiplex assay. **b–c.** Murine IC21 macrophages and PanIN neoplastic epithelial cells were incubated with 20ng/ml of rPEDF for 24 hours, and the culture media evaluated for IL8 by multiplex assay. **d.** PanIN/IC21 co-cultures were treated with 20ng/ml rPEDF for 24 hours, and the culture media analyzed for IL8 expression by ELISA. **e, f.** Serum samples from control and PEDF^−/−^ mice were analyzed for the levels of the anti-inflammatory cytokine IL10 and pro-fibrotic cytokine TGFβ1 by ELISA. (*, p<0.05).

To further evaluate the contributions of PEDF to augment an inflammatory cytokine profile, sera from control and PEDF^−/−^ mice were analyzed using a multiplex assay. While loss of PEDF affected the expression of several cytokines (Supplementary Figure S2), PEDF-null mice most notably displayed increased levels of the pro-inflammatory cytokine IL8 (Figure 5a). IL8 plays a regulatory role within the TME and is expressed by macrophages as well as epithelial cells [49]. Treatment of murine IC21 macrophages and PanIN cells with rPEDF *in vitro* suppressed IL8 production (Figure 5b, 5c). Moreover, PEDF prevented the gross overexpression of IL8 in the macrophage/epithelial co-cultures (Figure 5d). PEDF-null mice also demonstrated decreased levels of the anti-inflammatory cytokine IL10 (Figure 5e), further substantiating PEDF as an anti-inflammatory immunomodulator. Consistent with our observations *in vitro* (Figure 4c), PEDF-null mice exhibited increased expression of the pro-fibrotic cytokine TGFβ1 (Figure 5f).

### PEDF attenuates TGFβ-induced fibrosis

**Figure 6:**
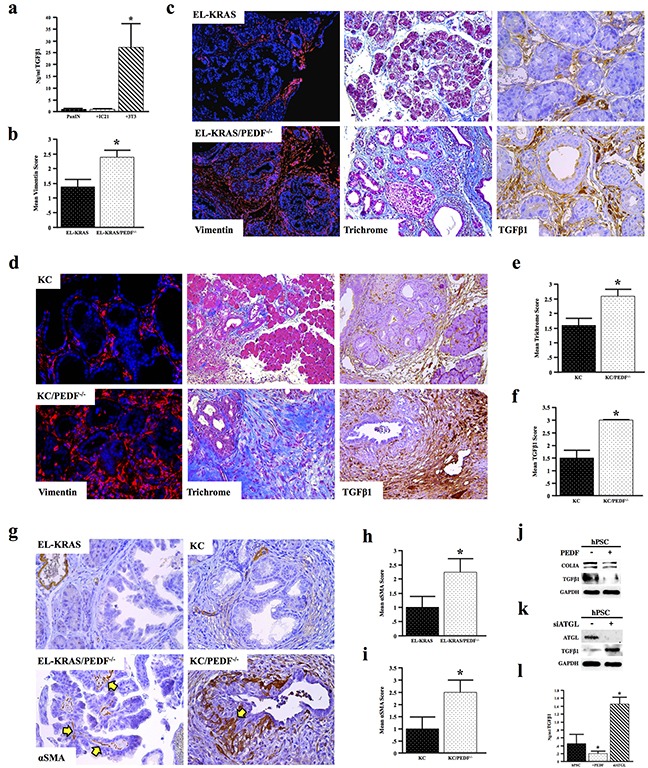
PEDF attenuates TGFβ-induced fibrosis **a.** Serum-free culture media generated over 24 hours from mouse PanIN neoplastic epithelial cells, 3T3 fibroblasts, and IC21 macrophages were analyzed for TGFβ1 level by ELISA. **b–c.** Tissue sections from age matched and EL-KRAS mice and EL-KRAS/PEDF^−/−^ mice were stained for vimentin, Mason's trichrome, or TGFβ1 and scored from 0-3+. **d–f.** KC and KC/PEDF^−/−^ mice were similarly stained for vimentin, Mason's trichrome, or TGFβ1 and scored as described previously. **g–i.** Pancreatic tumor sections from EL-KRAS, EL-KRAS/PEDF^−/−^ (n=4/group), KC, and KC/PEDF^−/−^ (n=3/group) mice were stained for αSMA, a marker of pancreas stellate cells, and slides scored. **j.** Human pancreatic stellate cells (hPSCs) were incubated with 20ng/ml rPEDF over 24 hours and the stellate lysates were subjected to Western blot analysis for COLIA and TGFβ1. **k.** ATGL was knocked down in hPSCs using siRNA (siATGL), and intracellular TGFβ1 expression measured by Western blot analysis. **l.** Culture media generated over 24 hours from control hPSCs, PEDF-treated hPSCs, and hPSCs transfected with siATGL were analyzed for secreted TGFβ1 by ELISA. (*, p<0.05). (6c top and middle bottom pictures at 200X 6c bottom right and left pictures at 400X 6d top pictures at 100X 6d bottom pictures at 200X 4g pictures at 200X).

Given the increase in serum TGFβ1 levels and the increased pancreatitis-associated fibrosis in PEDF null mice, we further evaluated the role of PEDF in regulating fibrosis. Initially, we evaluated the relative expression of the pro-fibrotic cytokine TGFβ1 in murine epithelial, myeloid, and mesenchymal cells. 3T3 murine fibroblasts expressed ∼20-fold more TGFβ1 than either the PanIN or IC21 cells, consistent with the stroma source of TGFβ1 in the tumor microenvironment (Figure 6a). We thus examined the effect of PEDF-deficiency on fibrosis and TGFβ1 expression *in vivo*. Significantly, both EL-KRAS/PEDF^−/−^ and KC/PEDF^−/−^ mice displayed increased fibrosis as determined by both vimentin and Mason's Trichrome staining, as well as increased TGFβ1 expression (Figure 6b–6f). EL-KRAS/PEDF^−/−^ and KC/PEDF^−/−^ mice also displayed increased α-smooth muscle staining (αSMA), consistent with an increase in activated PSCs (Figure 6g–6i). Furthermore, the PEDF receptor ATGL co-localized strongly with collagen IA, suggesting that the pancreatic stroma may respond to PEDF directly through ATGL (Supplementary Figure S3).

We therefore examined the effects of PEDF on human pancreatic stellate cells (hPSC) *in vitro*. hPSCs incubated with rPEDF displayed a moderate reduction in collagen IA deposition and a significant decrease in endogenous TGFβ1 (Figure 6j). Conversely, when the PEDF receptor ATGL was knocked down in hPSCs using siRNA, hPSCs displayed an increase in both endogenous as well as secreted TGFβ1 (Figure 6k, 6l), suggesting that PEDF may suppress TGFβ1 through the ATGL receptor and thereby modulate fibrosis *in vivo*.

### PEDF expression negatively correlates with fibrosis in human pancreatic cancer specimens

**Figure 7:**
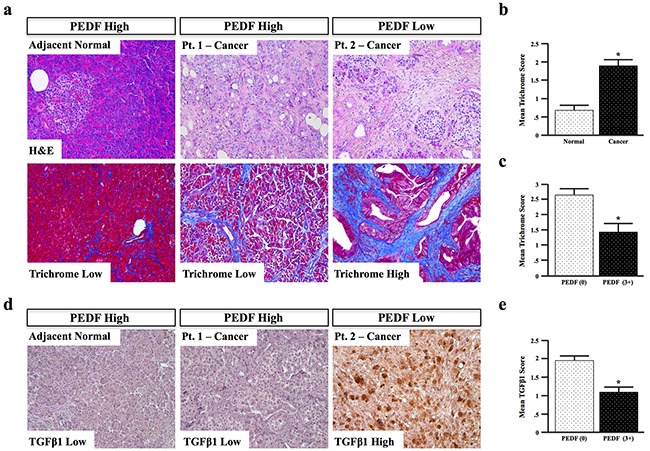
PEDF expression negatively correlates with fibrosis in human pancreatic cancer specimens **a–b.** Human pancreatic cancer and adjacent normal tissue sections (n=66) were stained via H&E and Mason's trichrome, and the extent of fibrosis scored from 0-3+. **c.** The correlation between PEDF and fibrosis was determined **d.** Tissue sections were stained for TGFβ1, and scored from 0-3+. **e.** The correlation between PEDF and TGFβ1 was also determined. (*, p<0.05). (All pictures with 20X objective.)

To provide additional *in vivo* support for our hypothesis that PEDF mitigates fibrosis, tissue sections from the same patient cohort used previously (Figure 1, N=66) were stained via H&E and Mason's trichrome, and fibrosis scored from 0-3+, also by two blinded investigators (representative images Figure 7a) As expected, the mean trichrome score was significantly higher in cancer specimens compared to adjacent normal tissue (Figure 7b). In accordance with our *in vitro* and *in vivo* observations, cancer sections with complete loss of PEDF had significantly higher fibrosis scores than groups with detectable or high PEDF expression (Figure 7c, p=0.026).

As PEDF suppressed expression of the pro-fibrotic cytokine TGFβ1 *in vitro*, we next assessed patients for expression of TGFβ1 and scored them as described above (representative images Figure 7d, N=66). Consistent with our *in vitro* observations, TGFβ1 expression was elevated in PEDF-deficient samples, and significantly (p<0.001) lower in PEDF-expressing samples (Figure 7e), further suggesting that PEDF suppresses TGFβ-mediated fibrosis in pancreatic cancer.

## DISCUSSION

Pancreatic cancer has a unique TME, characterized by a pronounced stromal reaction composed of collagen-rich extracellular matrix, pancreatic stellate cells, and inflammatory cells [18, 50] that contribute to hypovascularity [51] and hypoxia [52]. Though PEDF is a potent anti-angiogenic factor [53], pancreatic cancer-associated hypovasculatory and hypoxia are not likely mediated by PEDF, given that most patients have significantly reduced levels of PEDF. Furthermore, it has been suggested that vessel density does not appear to be a prognostic factor relevant to pancreatic cancer patients [54]. As PEDF expression correlates with favorable prognosis in PDAC [41], and disruption of PEDF signals contribute to the progression of pancreatic cancer, it is likely that PEDF loss may be affecting as yet an unidentified cell type in the TME through largely unknown cellular and molecular events [8–10, 12].

Based on our previous reports that loss of PEDF accelerates cerulein-induced pancreatitis and fibrosis [39] and mutant KRAS-induced pancreatic tumorigenesis [42], we hypothesized that PEDF may have tumor suppressor-like qualities. In pancreas, through anti-inflammatory and anti-fibrotic effects in non-epithelial cells. In human samples, we found that patients with PEDF deficiency indeed presented with both increased inflammatory cell involvement and tumor-associated fibrosis. These observations were substantiated in murine models of pancreatitis and carcinogenesis. Both displayed increased inflammation and enhanced fibrosis in the setting of more aggressive pancreatic cancer. Indeed, inflammation induced by PEDF loss is not unique to pancreatic cancer. There is a growing body of evidence demonstrating down-regulation of PEDF in several types of cancer, with rescue of PEDF associated with reduced tumor growth and improved animal survival. A variety of tumor-directed mechanisms were reported for the observed anti-tumorigenic properties of PEDF. These include induction of differentiation, pro-apoptotic effects (both cancer epithelial and endothelial cells), and direct inhibition of tumor cell invasion and migration [28]. Many of these may be linked to reduced levels of PEDF in the TME. In breast cancer, loss of PEDF correlated inversely with outcomes in patients, as re-expression of PEDF restored tamoxifen sensitivity in endocrine-resistant cancer cells, and loss of PEDF reduced tamoxifen sensitivity in endocrine-responsive cells [55]. Additional studies have shown that PEDF expression was also reduced in ovarian cancer patients, where exogenous PEDF inhibited the growth of both benign and cancerous ovarian cells [56]. In prostate cancer, PEDF was first shown to regulate both tumor mass and vascularity. The same study demonstrated the sufficiency of PEDF to limit prostate xenograft growth *in vivo* [33].

In this report, our findings suggest that PEDF opposes pancreatic cancer at least in part through anti-inflammatory and anti-fibrotic mechanisms. There is a growing body of literature on anti-inflammatory properties of PEDF in a variety of processes and tissues [26, 27]. This further extends our knowledge on the anti-tumorigenic properties of PEDF beyond its previously identified anti-angiogenic effects. A recent study showed that PEDF inhibition increases pro-inflammatory cytokines, macrophage infiltration, and inflammatory reactions in adipose tissues [57]. Similarly, PEDF has been implicated in countering the inflammatory changes in metabolic syndrome [58]. In diabetic retinopathy, PEDF suppresses retinal inflammation [59, 60] and once inhibited, leads to increased pro-inflammatory cytokine synthesis and macrophage infiltration [57]. In our study, the tumor inflammatory profile in the absence of PEDF was characterized by macrophage infiltration. This suggests an inhibitory role for PEDF on macrophage activity and recruitment. This was further supported by our *in vitro* experiments, where rPEDF neutralized migration of macrophages and enhanced mitogenic signaling in epithelial cells. While these results strongly suggest that PEDF negatively regulates inflammation, it has also been suggested that PEDF has pro-inflammatory effects [61]. Our observations seem to contrast findings in prostate cancer, where the anti-tumor property of PEDF was associated with higher macrophage recruitment [62]. This could be partially explained by tissue specific effects of PEDF, where its ability to recruit tumor-promoting or tumor-inhibitory macrophages depends on the context and tumor chronology. Indeed, it was observed in a prior study in prostate cancer that tumor macrophages recruited by PEDF initially possess cytotoxic anti-tumorigenic features. Eventually these macrophages suppress PEDF leading to lymph angiogenesis and tumor growth. Combined, these observations suggest that the immunomodulating effects of PEDF are highly tissue specific and warrant further study.

In human samples, we confirmed that PEDF is down-regulated in pancreatic cancer tissue and observed an inverse correlation between PEDF expression and tumor-associated inflammation and fibrosis. In two complementary *in vivo* mouse models of pancreatic cancer, lack of PEDF in KRAS^G12D^ mice enhanced myeloid cell infiltrations including macrophages and was associated with more aggressive pancreatic lesions. Consistent with PEDF's anti-inflammatory profile in cancer, loss of PEDF increased myeloid cell infiltration and overall inflammation, as evident in PEDF^−/−^ mice treated with cerulein. These findings suggest that PEDF may have the ability to attenuate KRAS^G12D^- and/or cerulein-induced inflammation in the pancreas. Likewise, recent evidence supports inflammation as being a key event in pancreatic cancer development [7, 63]. This is substantiated in the clinic, as it is well established that patients with a history of pancreatitis have a higher risk of developing pancreatic cancer [8, 10]. Furthermore, human pancreatic cancers have a robust immune cell infiltrate [6] and increased presence of inflammatory cells, such as mast cells. This has been linked to increased local and distant metastasis, worse tumor stage, and poor overall survival [5].

The mechanisms by which PEDF affects inflammatory-related signaling pathways have yet to be extensively studied. In human hepatocytes, PEDF ameliorates the effects of an IL1β challenge by suppressing activation of the inflammatory protein c-Jun N-terminal kinase (JNK) [58]. In prostate cancer cells, PEDF was shown to attenuate NFκB-mediated upregulation of IL8 [64]. IL8 is a pro-inflammatory cytokine that plays an important role in the progression of cancer in general [49] and pancreatic cancer in particular [65–67]. We found PEDF-null mice had higher serum levels of IL8. In two complementary *in vivo* mouse models of PDAC, genetic ablation of PEDF enhanced myeloid infiltration and was associated with more aggressive disease forms. *In vitro*, we demonstrated the sufficiency of rPEDF to suppress macrophage migration, NFκB expression, and synthesis of the pro-tumorigenic cytokine IL8 [65–67]. In a co-culture system, PEDF also suppressed macrophage stimulation of tumor-promoting mitogenic signals, including NFκB and IL8 in neoplastic epithelial cells. Further studies are necessary to identify signaling pathway(s) and cell type(s) mediating the anti-tumorigenic effects of PEDF in pancreatic cancer, highlighting the impact of PEDF on both parenchymal and mesenchymal cell compartments during pancreatic cancer progression.

An enhanced inflammatory response can lead to increased collagen deposition and fibrosis [68]. Although stroma has been recently suggested to potentially play a protective role in early stages of pancreatic carcinogenesis [69, 70], a dense stromal reaction parallels tumor progression and is commonly seen in resected specimens from pancreatic cancer patients [18]. Activated PSCs are the major mediators of a fibrotic TME [21, 71]. Upon activation, the PSCs begin expressing αSMA and depositing excess type I collagen. Activation of PSCs is dependent on growth factors, such as TGFβ [19, 21], and clinical studies demonstrate an elevation in TGFβ signaling in human PDAC tumor tissue, especially in the areas of fibrosis [22–25]. We found that increased fibrosis seen in the absence of PEDF in mice or humans was associated with enhanced TGFβ1 expression. This subsequently led to an increase in PSCs, which were responsive to rPEDF and showed a reduction in TGFβ1 and collagen IA deposition. These effects may be exerted, at least in part, via the PEDF receptor ATGL in hPSCs. This is consistent with recent studies showing the anti-fibrotic effects of PEDF in the liver, partly through reduced activity of stellate cells [72, 73]. In other tissues, PEDF also was reported as an anti-TGFβ and anti-fibrosis factor [74, 75], which supports our findings of increased TGFβ1 levels in the serum of PEDF-null mice. Contrary to our results, PEDF was found to activate hPSCs as measured by αSMA expression, though TGFβ was not assessed [38]. These contradictory observations may be due to differences in the source of tested PSCs, the differences in culture conditions, and limitations associated with *in vitro* assays. However, similar to our findings and those from others (19), the authors also found that PEDF associates with more favorable measures in patients with PDAC. These observations could be better explained by our data showing the inhibitory effect of PEDF on PSC activation, collagen deposition, and tumor-associated fibrosis and inflammation. This is further increased in the absence of PEDF in two established *in vivo* models of PDAC and one of pancreatitis.

While little is known about PEDF and its many contributions to carcinogenesis, PEDF emerges as a critical factor with clear tumor suppressor properties in pancreatic cancer, relaying signals between the TME and cancer cells. Our findings extend a therapeutic role for PEDF in pancreatic cancer, particularly by targeting stromal and hematopoietic cell compartments to mitigate tumor progression.

## MATERIALS AND METHODS

### Cell lines, co-cultures, and migration assays

Murine IC21 macrophages were maintained in Roswell Park Memorial Institute (RPMI) 1640 media supplemented with 10% heat-inactivated fetal bovine serum (FBS), penicillin (100 units/μL), and streptomycin (100μg/mL). MD human macrophages, human pancreatic stellate cells (hPSC), and mouse neoplastic cells (PanIN) were cultured in Dulbecco's Modified Eagle Medium (DMEM) supplemented with 10% heat-inactivated fetal bovine serum (FBS), penicillin (100 units/μL), and streptomycin (100 μg/mL). All cells were cultured in a 37°C incubator with 5% CO_2_.

Co-cultures were established by seeding PanIN epithelial cells in the bottom of 6 well plates, and species-corresponding IC21 macrophages in transwell inserts in separate plates. Cells were allowed to adhere in their own media for 24 hours, then the stroma containing transwell inserts were added to the 6 well plates containing the epithelial monolayers. Cells were given fresh media, allowed to acclimate for 24 hours, and starved of serum overnight prior to TGFβ treatment.

Migration assays were performed in 6 well plates by seeding macrophages in transwell chambers in serum free media. A chemoattractant (10% FBS) was introduced to the bottom chamber. After 24 hours, transwell chambers were removed and non-migrating cells were removed with a cotton swab. Membranes were removed from transwell chambers, Geimsa stained, mounted, and migrating cells quantified per 10x field.

### siRNA transfections

ATGL siRNA (Invitrogen, Grand Island, NY) was reconstituted in nuclease free water per manufacturer specification, and delivered at 3nM in RNAiMAX transfection reagent (Invitrogen). Knockdown was validated via western blotting.

### Chemicals and reagents

rPEDF was generated by transfection of an rPEDF vector into baby hamster kidney (BHK) cells and used at 20ng/ml.

### Mice

EL-KRAS [76] and PEDF^−/−^ [42] mice were previously generated and characterized as described. Cohorts of C57BL/6 control, PEDF^−/−^, EL-KRAS, Pdx1-Cre/LSL-KRAS^G12D^ (KC) [77], EL-KRAS/PEDF^−/−^, and KC/PEDF^−/−^ mice were euthanized at time points between six months and one year.

Ten to twelve week-old mice (PEDF^−/−^ and control) were given 6 hourly intra-peritoneal injections of either supra-physiological levels of cerulein (50 μg/kg) or PBS. An hour after the last injection, mice were anesthetized using ketamine/xylazine (100/10 mg/kg) after which blood was collected using cardiac puncture.

### Western blot

Cell or tissue lysates were lysed in 4% SDS buffer followed by needle homogenization. Equal amounts of protein (15-40 μg) were mixed with loading dye, boiled for 8 min, separated on a denaturing SDS-polyacrylamide gel and transferred to a PVDF membrane. The membrane was blocked in 5% milk/TBS/0.1% Tween for 1h and incubated with antibodies against ADAM17, NFκB, p21, GAPDH (Santa Cruz Biotechnology, Dallas, TX), Collagen IA (Southern Biotech, Birmingham, AL), TGFβ1 (abcam, Cambridge, MA), pSTAT3, pERK, pAKT (Cell Signaling, Danvers, MA), or ATGL (eBioSci, San Diego, CA).

The membrane was washed with TBS-0.1% Tween and then incubated with HRP-conjugated secondary antibody (Santa Cruz) at room temperature for 1h and rewashed. Protein bands were visualized by an enhanced chemiluminiscence method (Thermo, Waltham, MA) and resolved digitally per the manufacturer's specifications.

### Histology, immunohistochemistry, and immunofluorescence

Age-matched control, PEDF^−/−^, EL-KRAS, KC, EL-KRAS/PEDF^−/−^, and KC/PEDF^−/−^ mice were euthanized and subjected to pathological examination the pancreas, colon, small bowel, liver, and spleen. Tissues were fixed in 10% formalin, paraffin-embedded, and sections at 4μm interval were cut from each tissue and stained with H&E, Mason's trichrome (Sigma Aldrich), or via immunohistochemistry (IHC)/immunofluorescence (IF). Esterase staining using Chloracetate Esterase (CAE) stain was done to detect myeloid cells (e.g., granulocytes as well as monocytes/macrophages) [46, 78]. For CAE staining, slides were deparaffinized by xylenes and rehydrated by ethanol gradient. New Fuchsin, 4% Sodium Nitrite, Phosphate Buffer, and Naphthol AS-D Chloroacetate were combined over ice and applied to tissue sections for 20 minutes. Slides were rinsed and counterstained with hematoxylin before mounting.

For IHC, after deparaffinization/rehydration, slides were heated in a pressure cooker using DAKO retrieval buffer. Endogenous peroxidases were quenched in 3% hydrogen peroxide in methanol for 30 min. Tissues were blocked with 0.5% BSA in PBS for 30 min and exposed to primary antibodies against, pERK (Cell Signaling), PEDF, TGFβ1 (Abcam), and NFκB (Santa Cruz). Slides were developed using species-specific secondary antibodies, followed by DAB substrate/buffer (DAKO, Carpinteria, CA).

For IF, after deparaffinization/rehydration, slides were heated via pressure cooker in DAKO retrieval buffer and tissues blocked with 5% donkey serum in PBS for 1 hour at room temperature. Sections were exposed to primary antibodies against CK19 (University of Iowa Hybridoma Bank), ATGL, F4/80 (eBiosSci), PCNA, αSMA, CD11b (Abcam), COLIA (Southern Biotech), and Vimentin (Cell Signaling) at 1:50-1:200 overnight at 4°C. Slides were developed using Alexaflor 488 or 594 conjugated secondary antibodies (1:200-1:500, Abcam), mounted in DAPI containing media (Santa Cruz), exposed to DAPI, FITC, and Texas Red filters, and images superimposed.

Staining intensity was determined by two blinded and independent investigators. Tissues with undetectable expression were scored as 0, and tissues with strong, ubiquitous expression scored 3+. For sections with intermediate staining, scores of 1-2+ were assigned based on the expertise of the blinded investigators based on variance from 0 and 3+.

### ELISA

Affymetrix Human/Mouse TGFβ1 ELISAs were purchased and used per manufacturer specification.

### Statistical analysis

Data were analyzed by two-way ANOVA and fit to a general linear model in Minitab16, the validity of which was tested by adherence to the normality assumption and the fitted plot of the residuals. Results were arranged by the Tukey method, and were considered significant at p<0.05.

Minitab 16 also was used to run two-tailed T tests where appropriate. *In vitro* results are presented as ± S.D., and *in vivo*/clinical results are presented as mean ± S.E.M unless otherwise noted.

### Study approval

All experiments involving the use of mice and human subjects were performed following protocols approved by either the Institutional Animal Care and Use Committee or local IRB at the Northwestern University. Patient slides and information was obtained in a de-identified fashion from the Northwestern University Pathology Department.

## SUPPLEMENTARY FIGURES


