# Supplementary material for: PEDF inhibits pancreatic tumorigenesis by attenuating the fibro-inflammatory reaction
Source: Oncotarget. 2016 Apr 5;7(19):28218–34. doi: 10.18632/oncotarget.8587 (PMC5053722; doi:10.18632/oncotarget.8587)
Supplement: Supplementary file 1 [file oncotarget-07-28218-s001.pdf]

SUPPLEMENTARY FIGURES

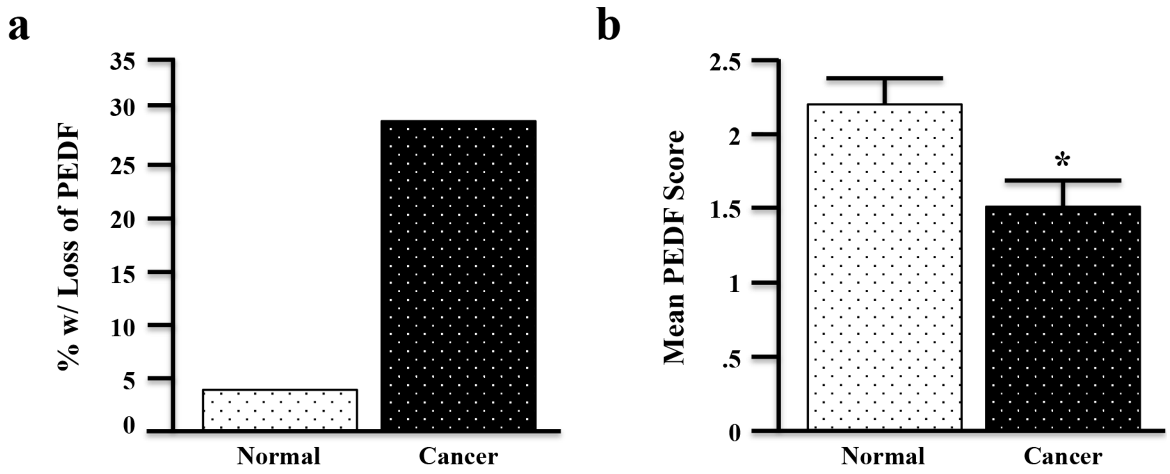

Supplementary Figure S1: PEDF Expression is Reduced in Primary Pancreatic Cancer Samples. a. Human pancreatic cancer and non-malignant tissue sections were stained for PEDF and scored from 0-3+. Approximately 30% of cancer tissue had complete loss (0 score) of PEDF expression, while this was observed in less than 5% of adjacent, non-malignant sections. **b.** Cancer tissues had significantly reduced mean PEDF expression compared to adjacent normal sections. (*, $p<0.05$).

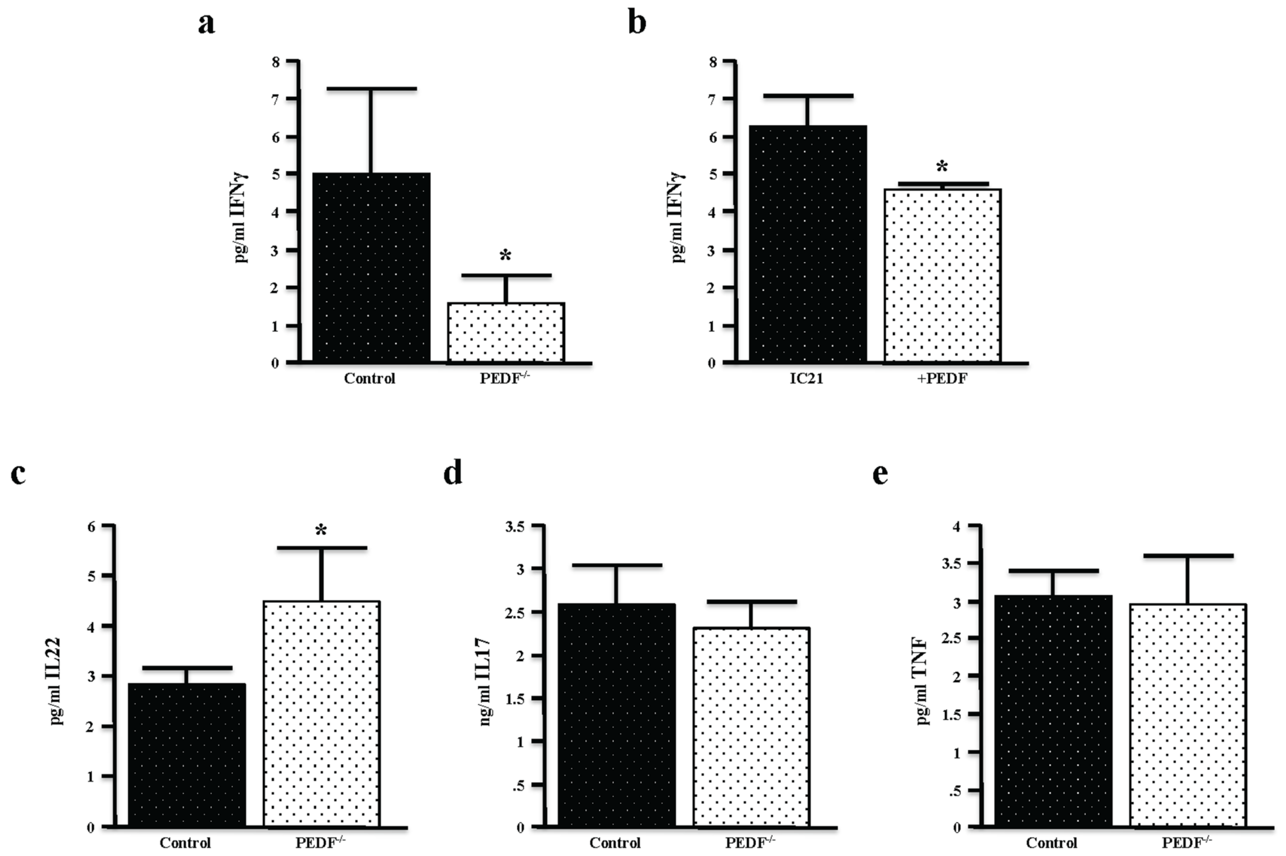

Supplementary Figure S2: Loss of PEDF Increases Pro-Inflammatory Cytokines. a. Serum samples from four-month-old control and PEDF$^{-/-}$ mice were evaluated for expression of the anti-proliferative cytokine interferon-γ (IFNγ). **b.** Murine IC21 macrophages were incubated with 20ng/ml of rPEDF for 24 hours, and the culture media evaluated for IFNγ by multiplex assay. **c–e.** Serum from control and PEDF$^{-/-}$ mice were next evaluated for expression of the inflammatory cytokines IL22, IL17, and TNF by multiplex assay. (*, $p<0.05$).

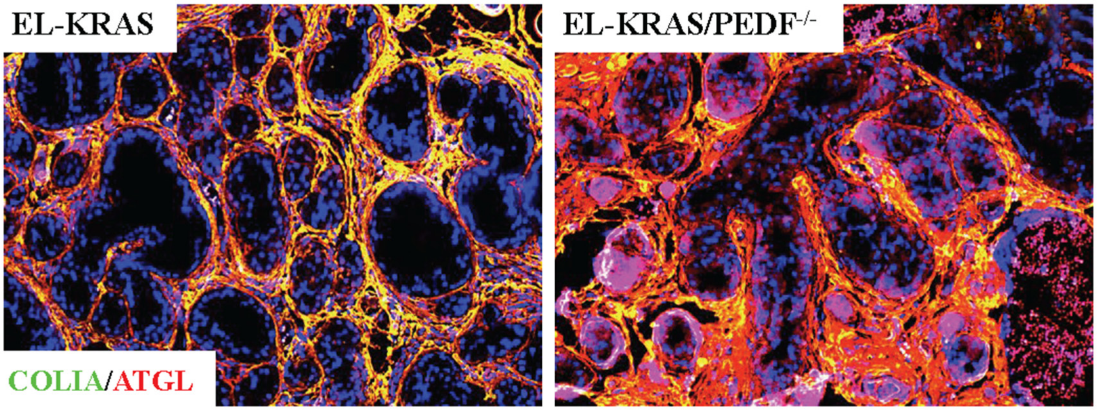

Supplementary Figure S3: ATGL is Expressed in the Pancreatic Stroma. Pancreatic tumor sections from control EL-KRAS mice (left) and EL-KRAS/PEDF$^{-/-}$ (right) mice were dual-stained with anti-Collagen IA and anti-ATGL.
